# Comparative analysis of diabetes risk in patients with obstructive sleep apnea undergoing different treatment approaches

**DOI:** 10.1186/s40463-023-00651-8

**Published:** 2023-08-11

**Authors:** Nai-Chen Shih, James Cheng-Chung Wei

**Affiliations:** 1 Department of Family Medicine, Taichung Veterans General Hospital, 1650 Taiwan Boulevard Sect. 4, Taichung, 40705 Taiwan, ROC; 2 Institute of Medicine, Chung Shan Medical University, Taichung, Taiwan; 3 Department of Allergy, Immunology and Rheumatology, Chung Shan Medical University Hospital, 110 Jianguo N Rd, Section 1, South District, Taichung, 40201 Taiwan; 4 Graduate Institute of Integrated Medicine, China Medical University, Taichung, Taiwan; 5 Department of Medical Research, Taichung Veterans General Hospital, Taichung, Taiwan

**Keywords:** Obstructive sleep apnea, Diabetes mellitus, Continuous positive airway pressure, Surgery

## Abstract

The bidirectional relationship between obstructive sleep apnea (OSA) and diabetes mellitus (DM) has been explored in several studies. O’Connor-Reina et al., published a paper entitled: “Risk of diabetes in patients with sleep apnea: comparison of surgery versus CPAP in a long-term follow-up study” to examine a cohort study comparing the effects of surgery and continuous positive airway pressure (CPAP) on the risk of diabetes in patients with OSA. The study findings suggest that both therapies offer protection against diabetes, with upper airway surgery demonstrating better preventive efficacy than CPAP. However, potential biases and confounding variables should be considered, such as race, ethnicity, socioeconomic factors, BMI, glucose levels, HbA1c values, medication use and healthy dietary habits. Besides using International Classification of Diseases codes, the definition of DM as an outcome can also incorporate specific laboratory indicators and the use of diabetes treatment medications. Furthermore, subgroups analysis defined by demographic variables, such as age, sex, and race is recommended. The limitations of the study also include potentially data omissions due to reliance on electronic medical records from specific healthcare institutions. To enhance research comprehensiveness, alternative data sources and collaborations with additional healthcare institutions are suggested for future investigations.

## Main text

Previous studies have investigated a bidirectional relationship between obstructive sleep apnea (OSA) and diabetes mellitus (DM). The potential intermediary mechanisms linking OSA to T2DM involve alterations in the HPA axis, increasing oxidative stress, sympathetic activation and activating inflammatory pathways [1].

The selection of the most suitable treatment approach for OSA depends on individual patient characteristics, preferences, and the severity of the condition. Continuous positive airway pressure (CPAP) is a well-established and mainstay therapeutic approach for managing OSA. However, in certain cases, physicians may recommend alternative options include oral appliances, various surgical procedures, modifying risk factors, such as weight loss.

With great interest, we read the cohort study from O’Connor-Reina et al. [2], published a paper entitled: “Risk of diabetes in patients with sleep apnea: comparison of surgery versus CPAP in a long-term follow-up study”. The authors found that both therapies protect against diabetes. Additionally, upper airway surgery can prevent the development of diabetes better than CPAP. The author's arguments are compelling and supported by evidence, yet it is crucial to recognize and mitigate any potential biases that could have affected the interpretation of the data.

First, the primary outcome of this study was development of new-onset diabetes. The cohorts were matched on comorbidities and demographic characteristics including age and sex. However, there were still some factors that were not regarded as confounding variables and thus were not taken into consideration. Owing to the fact that this study was conducted with data obtained from TriNetX, a global federated health research network, the factors of race and ethnicity need to be taken into account. Also, the database enables researchers to explore the interplay between socioeconomic factors and health outcomes. In spite of matching comorbidities with specific ICD-10 codes for overweight, BMI can still be included as a contributing factor. Other vitals and key laboratory findings: glucose (mg/dL) levels and HbA1c values are also suggested as study factors. Moreover, medications such as long-term use of glucocorticoids, beta-blockers, thiazide diuretics or certain antidepressants are often considered as covariates to control for their impact on diabetes outcomes and determine the independent effects of other factors on diabetes. To our knowledge, important measures to reduce diabetes risk are tobacco cessation, quitting drinking. However, these measures were not considered as confounding factors in this research.

Second, the main outcome “Diabetes” was recorded with ICD codes in this study. The assignment of ICD codes is usually performed by healthcare professionals and involves a certain degree of subjectivity. Therefore, besides using ICD codes to define DM as an outcome in research, there are other definition methods. Typically, specific laboratory indicators such as blood glucose levels, glycated hemoglobin (HbA1c) thresholds are used to determine the presence of diabetes. In addition, diabetes can be defined based on the use of diabetes treatment medications.

Third, subgroups analysis defined by demographic variables, such as age, sex, and race is recommended. A significant association has gender difference between the severity of AHI, severe hypoxemia and blood glucose [3]. Besides, in another study, African individuals with OSA exhibit a distinct comorbidity profile compared with Caucasians. African group comprises younger patients who have a higher prevalence of diabetes [4].

Finally, the authors present an excellent summary of the association between different OSA treatment approaches and new-onset diabetes in patients with OSA while highlighting study limitations due to the use of electronic medical records as the data source. The reliance on patient records from specific healthcare institutions and clinical research centers in the TriNetX database may result in data omissions if a patient seeks treatment (ex: surgery) at a non-connected hospital. This limitation can affect the comprehensiveness and representativeness of the research. Alternative data sources or collaborations with other healthcare institutions may be needed to address this limitation and achieve broader data coverage.

## Data Availability

The datasets supporting the conclusions are included within the article.

## Abbreviations

OSA: Obstructive sleep apnea
DM: Diabetes mellitus
CPAP: Continuous positive airway pressure
HPA axis: Hypothalamic–pituitary–adrenal axis
BMI: Body mass index
ICD: International Classification of Diseases
AHI: Apnea–hypopnea index
